# Photo-triggered large mass transport driven only by a photoresponsive surface skin layer

**DOI:** 10.1038/s41598-020-69605-8

**Published:** 2020-07-29

**Authors:** Issei Kitamura, Keisuke Kato, Rafael Benjamin Berk, Takashi Nakai, Mitsuo Hara, Shusaku Nagano, Takahiro Seki

**Affiliations:** 1 Department of Molecular and Macromolecular Chemistry, Graduate School of Engineering, Nagoya University, Furo-cho, Chikusa, Nagoya 464-8603 Japan; 2 Department of Chemistry and Catalysis Research Center, Technical University of Munich, 85748 Garching, Germany; 3 Department of Chemistry, College of Science, Rikkyo University, 3-34-1 Nishi-Ikebukuro, Toshima-ku, Tokyo, 171-8501 Japan

**Keywords:** Liquid crystals, Polymers

## Abstract

Since the discovery 25 years ago, many investigations have reported light-induced macroscopic mass migration of azobenzene-containing polymer films. Various mechanisms have been proposed to account for these motions. This study explores light-inert side chain liquid crystalline polymer (SCLCP) films with a photoresponsive polymer only at the free surface and reports the key effects of the topmost surface to generate surface relief gratings (SRGs) for SCLCP films. The top-coating with an azobenzene-containing SCLCP is achieved by the Langmuir–Schaefer (LS) method or surface segregation. A negligible amount of the photoresponsive skin layer can induce large SRGs upon patterned UV light irradiation. Conversely, the motion of the SRG-forming azobenzene SCLCP is impeded by the existence of a LS monolayer of the octadecyl side chain polymer on the top. These results are well understood by considering the Marangoni flow driven by the surface tension instability. This approach should pave the way toward in-situ inscription of the surface topography for light-inert materials and eliminate the strong light absorption of azobenzene, which is a drawback in optical device applications.

## Introduction

Surface morphology generations on azobenzene(Az)-containing polymer films induced by patterned irradiation have been the active area in photofunctional material research. In 1995, Natansohn’s^1^ and Tripathy’s^2^ groups independently reported the induction of surface relief gratings (SRGs) on Az amorphous films using interference irradiation of two laser beams. Since then, photoinduced SRG processes have been reported using various photofunctional materials such as amorphous polymers^1–20^, side chain liquid crystalline polymers (SCLCPs)^21–27^, supramolecular systems^24,27–29^, amorphous molecular materials^30^. Additionally, SRG formation can be realised using other photoresponsive units^31–35^.

Various mechanistic models for mass transfer have been proposed: isomerisation pressure due to volume change^7,8^, gradient force^9–11^, mean-field model^12^, directed softening or fluidisation^13–15^, molecular diffusion^16–18,34^, molecular orientation force^19,20^, etc. With regard to Az-containing SCLCPs^21–27^, UV light irradiation leads to a photochemical phase transition between the liquid crystal (LC) and isotropic phases. This phase change plays an important role in highly efficient SRG formation^23,25,26^, which requires an overall dose of much less than 1 J cm^–2^. Typically mass transport depends on the light irradiation conditions and physicochemical properties. Consequently, a universal explanation about the mechanism has yet to be provided.

Recent studies have noted the importance of the surface effect on the mass transfer process. Ambrosio et al.^17,18^ proposed an anisotropic light-driven molecular diffusion model for spiral morphology induction under vortex-beam illumination. Their model stressed enhanced molecular diffusion in proximity of the free surface. Ellison et al.^36–39^ have proposed microfabrications via the Marangoni flow by photochemical reactions. Similarly, we reported UV light-induced mass transfer of an Az-containing SCLCP film at the inkjet-printed lines of another polymer^40^. In this case, mass transfer could be explained by the Marangoni flow occurring at the inkjet-printed areas. Related to the molecular orientation in SCLCP films, an Az-containing command layer at the free surface or inkjet-printed patterns could control the orientation of the majority of the mesogens of the base film^41–45^. Kawatsuki et al.^46,47^ also controlled of mesogen orientation from free surfaces using different types of photoreactive units.

**Figure 1:**
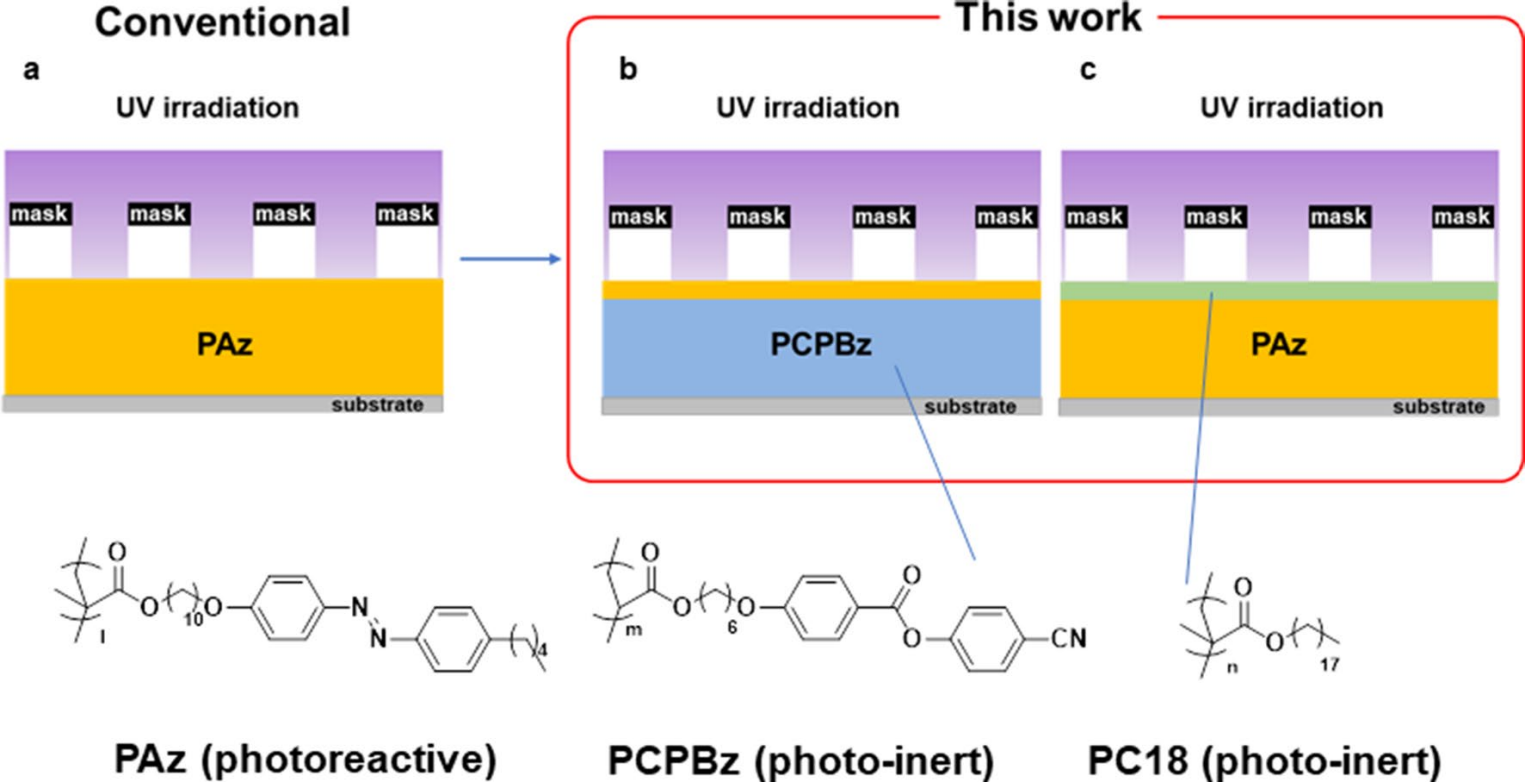
Schematic of photo-triggered mass transport systems and the chemical structures of the polymers. Conventional type contains Az units scattered in the entire films (**a**), light-inert films with a photoreactive surface (**b**), and photoactive films with a light-inert surface (**c**).

The above knowledge inspired us to examine light-driven mass transport for “light-inert” SCLCP films with only a photoresponsive layer at the free surface and a photoresponsive SCLCP films covered with a light-inert molecular layer. Such a study should provide decisive evidence to extract the effect of the topmost surface. Figure 1 schematically depicts the materials and systems used in this work. This study demonstrates that the existence of a nm-thick photoresponsive skin layer on the surface can induce mass transfer (Fig. 1b). Conversely, the existence of 1–2-nm-thick layer of the octadecyl side chain polymer on the top of the film almost fully hinders transport motions (Fig. 1c). These observations highlight the critical role of the topmost surface in the macroscopic mass transfer of polymer films. Moreover, they suggest that previous arguments in SRG studies should be reconsidered. Finally, this work demonstrates the technological importance in the microfabrication of materials without photoreactive units.

## Results and discussion

### Polymer synthesis

Az-containing, cyanophenyl-benzoate containing, and octadecyl methacrylate polymers (PAz^40,42^, PCPBz^48^, and PC18^49^, Fig. 1) were synthesised from their corresponding monomers by atom transfer radical polymerisation (ATRP). The molecular mass and the molecular mass distribution were measured by gel permeation chromatography or high-performance liquid chromatography (Showa Denko, Japan). The liquid crystal transition temperatures and glass transition temperatures were evaluated by differential scanning calorimetry (DSC, TA Instrument Q200, USA) and polarised optical microscopy (BX-51, Olympus, Japan). Figure S1 shows the DSC curves. The thermal phase transition behaviour of PAz is reported elsewhere^40,42^. PCPBz exhibited a nematic phase between 23–114 °C (Fig. S2).

Table 1 summarises the molecular mass data and thermophysical properties of the synthesised polymers.

**Table 1:** Synthesis and characterisations of the polymers used in this study.

Polymer	M_n_ (M_w_/M_n_)	Degree of polymerisation	Thermophysical properties/°C	*θ*_gly_/°	
				25 °C	90 °C
PAz	1.4 × 10^4^ (1.08)	26	g (45) sm C (87) sm A (110) iso	102.5 ± 0.9	103.2 ± 0.7
PAz (UV)				89.1 ± 1.8	85.1 ± 1.6
PCPBz	5.1 × 10^4^ (1.19)	129	g (23) nematic (114) iso	76.1 ± 1.0	80.1 ± 1.5
PC18	3.4 × 10^4^ (1.13)	88	cryst (27) iso	107.1 ± 0.9	

*UV* under UV light irradiation, *g* glass, *sm* smectic, *iso* isotropic, *cryst* crystal.

### Contact angles of polymer films

Table 1 lists the *θ*_gly_ values. For reference, *θ*_w_ on the polymer films at 25 °C are shown in Fig. S3a. The *θ*_gly_ values on PAz were 102.5 ± 0.9° (25 °C) and 103.2 ± 0.7° (90 °C), but decreased to 89.1 ± 1.8° (25 °C) and 85.1 ± 1.6° (90 °C) under UV irradiation, respectively. For all temperatures, the *θ*_gly_ values of unirradiated PAz were larger than those under UV light irradiation, indicating that UV irradiation induces a higher surface tension on the PAz surface as the cis-isomers of Az increases^50,51^. We previously reported that the cis-Az content under UV light irradiation at 1.0 mW cm^–2^ reaches 70–80% at 80 °C^25^. The cis-Az content at 90 °C should be slightly smaller than this. Moreover, the *θ*_gly_ values for light-inert PCPBz were 76.1 ± 1.0° (25 °C) and 80.1 ± 1.5° (90 °C), demonstrating that the light-inert PCPBz has a substantially higher surface tension. On the other hand, the surface tension of PC18 was low as *θ*_gly_ = 107.1 ± 0.9° at 25 °C. These values were used to characterise the topmost surfaces.

### PCPBz films with a PAz LS layer

**Figure 2:**
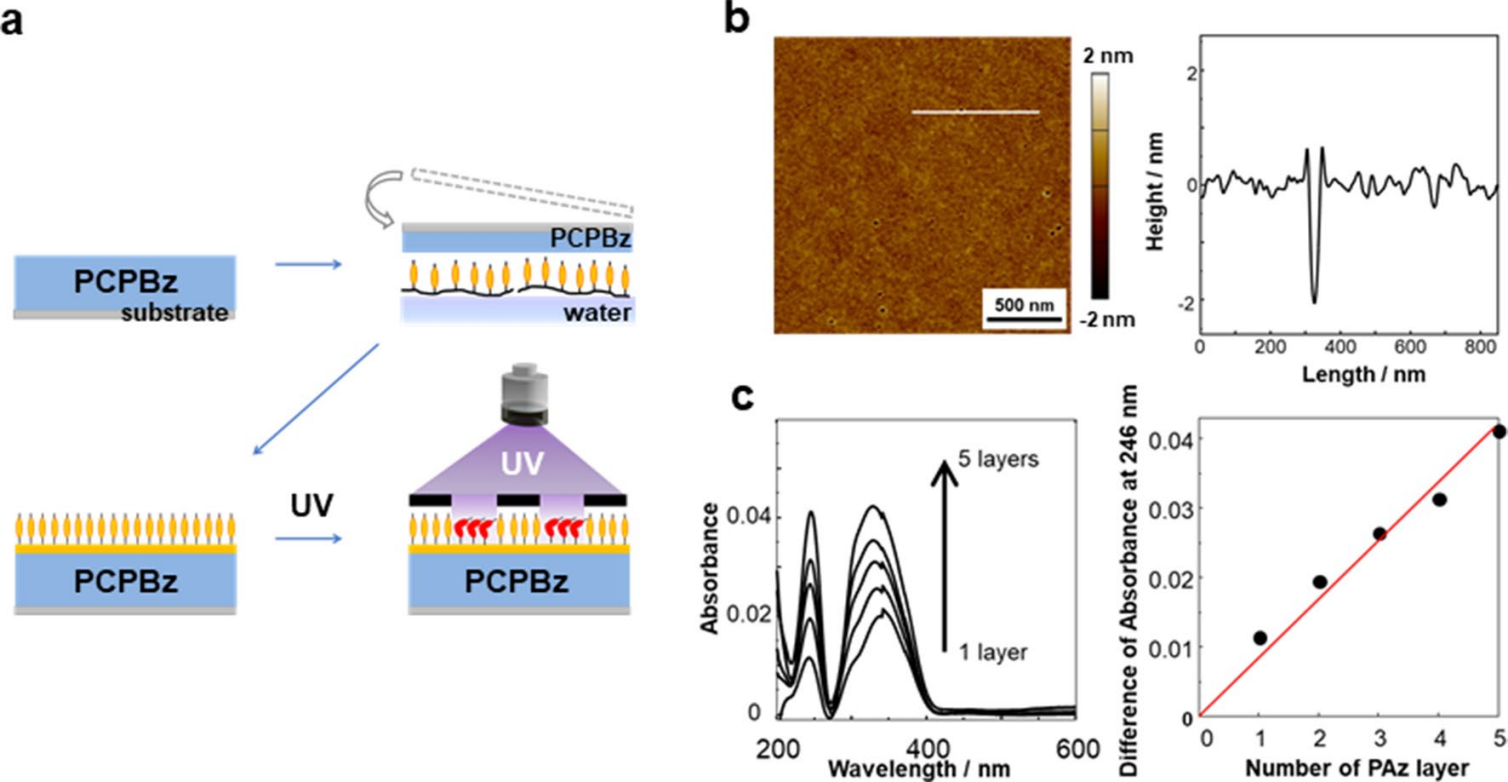
LS deposition of a PAz monolayer on a PCPBz film. Schematic of LS deposition of a PAz layer on a PCPBz film and successive UV irradiation through a stripe mask (pitch: 20 µm) (**a**). Topographical atomic force microscopy (AFM) images of a PAz monolayer film on Si wafer (**b**, left) and cross-sectional profile obtained from the AFM data (**b**, right). UV–visible absorption spectra for different deposition numbers of PAz layers (1–5 layers) on the PCPBz film (**c**, left) and the absorbance of PAz at 246 nm as a function of the deposition number of PAz monolayers (**c**, right).

Figure 1b shows the light-inert SCLCP covered with a photoactive layer. Repeated deposition of a PAz floating monolayer by the LS method realised a molecularly controlled coating onto the surface of PCPBz films (Fig. 2a). Figure S4 shows the pressure-surface area isotherm (*π*-*A* curve) of PAz on the water surface at 20 °C. Based on the steep uprise in the π-*A* curve, the estimated molecular occupied area of the Az monomer unit was 0.3 nm^2^. This value agrees well with that previously reported for a similarly occupied area of mesogenic Az polymers^52,53^, suggesting that PAz forms a stable monolayer film on a water surface.

The PAz monolayer was transferred onto a quartz substrate at a pressure of 15 mN m^−1^. Figure 2b shows the topographical AFM image (left) and a height profile (right) of PAz monolayer on a hydrophobised Si wafer with 1,1,1,3,3,3-hexamethydisilazane (left). The estimated thickness of the PAz monolayered film at a defect area was 2 nm.

Figure 2c displays the UV–visible absorption spectra of the PAz layers as a function of the number of deposition layers on the PCPBz film after subtracting the spectrum of the pure PCPBz film. There were two absorption bands at 246 nm (*ϕ*–ϕ* transition) and 348 nm (*π*–π* transition) of the Az unit. The *ϕ*–ϕ* absorption at 246 nm increased proportionally with the deposition number, indicating that the PAz layer is accurately deposited onto the PCPBz film. This band was chosen to check the deposition state because the transition moment is independent of the Az orientation.

**Figure 3:**
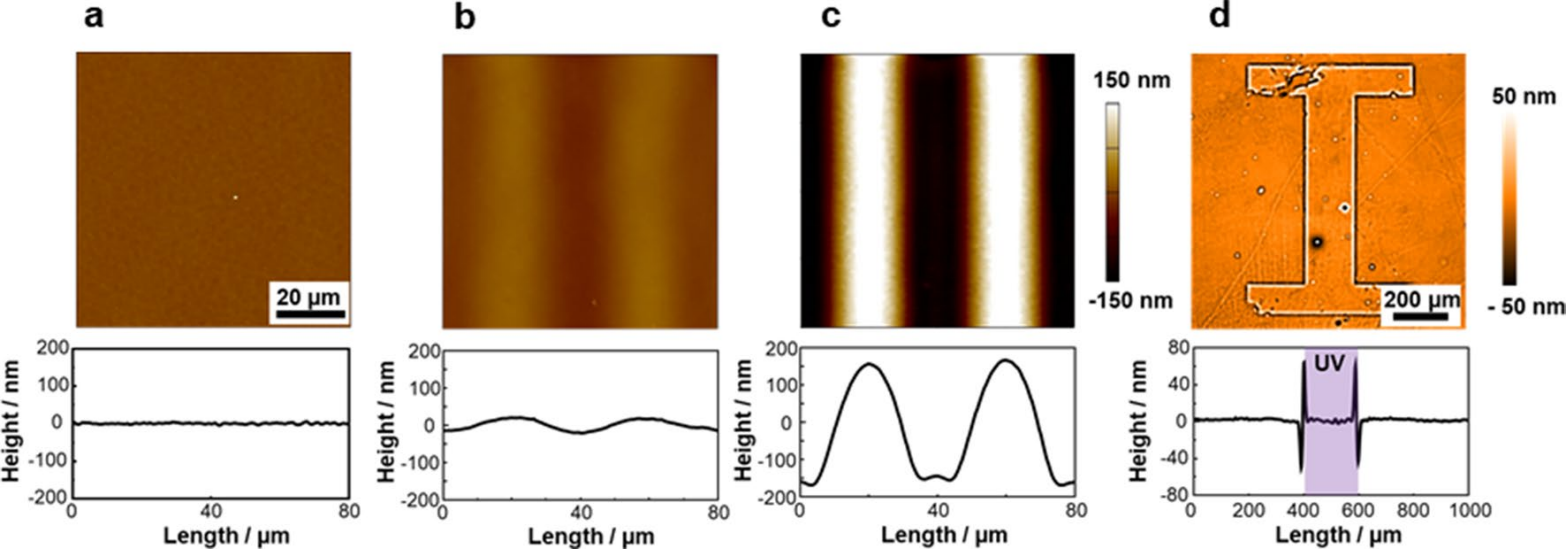
Photoinduced mass migration behaviour of PCPBz films with the LS layer of PAz. Topographical AFM images (upper) and cross-sectional height profiles (lower) after stripe-patterned UV irradiation at 90 °C on PCPBz films without a PAz layer (**a**), with a 1-layered (**b**), and 10-layered (**c**) LS film of PAz on the surface of 200-nm-thick PCPBz film. (**d**) Topographical WLIM image (upper) cross-sectional height profile (lower) of a PCPBz film with a 5-layered PAz LS film at the surface after UV irradiation through a masked shaped like the letter “I”.

Next, AFM topographical images were acquired for 200-nm thick PCPBz films covered with the PAz LS layer. Figure 3 displays the height profiles after UV light irradiation (1.0 mW cm^–2^ for 300 s) at 90 °C through a line and space photomask (20-µm pitch). The temperature control is an important factor in the SCLCP systems^25^, and the most efficient mass migration occurred at 90 °C in the present case (Fig. S5). A pure PCPBz film had a flat surface, and UV irradiation did not induce a topological change (Fig. 3a). However, depositing a PAz LS layer on the PCPBz film caused obvious surface undulations. Even a 2-nm-thick PAz monolayer deformed the PCPBz film surface. The height difference between the peak and valley induced by the PAz monolayer was 41 nm (Fig. 3b), demonstrating that a 2-nm-thick PAz monolayer on the surface can generate a SRG with ca. 20-times larger surface undulation. The 10-layered PAz resulted in a top-to-bottom difference of 334 nm (Fig. 3c). Hence, a thicker top-coated PAz layer resulted in a larger deformation. For the 10-layered PAz film, the mass motion became saturated where the substrate surface was almost exposed (Fig. 3c).

Since the protrusions and the trenches preserves the polymer volume, this surface topography originates from a mass transfer. To evaluate the mass flow direction, we adopted a photomask with an “I”-shaped transparent window. Figure 3d shows the surface topographical image and height profile extracted along the line for a 5-layered PAz/PCPBz film observed with a white light interference microscopy (WLIM). Mass transfer occurred at the boundary region between the UV light irradiated and the non-irradiated areas. The height positions of UV-exposed and shaded areas were higher and lower, respectively. Hence, lateral mass flow occurred from the trans-Az area to the cis-rich-Az one of the PAz skin layer at the surface. The cis-Az surface had a larger wettability for polar solvents^50,51^, as confirmed by the smaller values of *θ*_gly_ throughout the examined temperature range (Fig. S3b). Thus, PAz in the cis-rich state has a higher surface tension than that in the trans state. These results agree with the reports by Ellison et al.^36^ for the surface fabrication by photopatterning onto an Az polymer and our observations in inkjet printed system^40^. The effect of linear UV light polarisation was examined. As shown in Fig. S6, a polarisation dependence was not observed^54^ for both the PAz-covered PCPBz film and a pure PAz film. This fact also suggest that the observed mass transport is attributed to the Marangoni flow mechanism.

**Figure 4:**
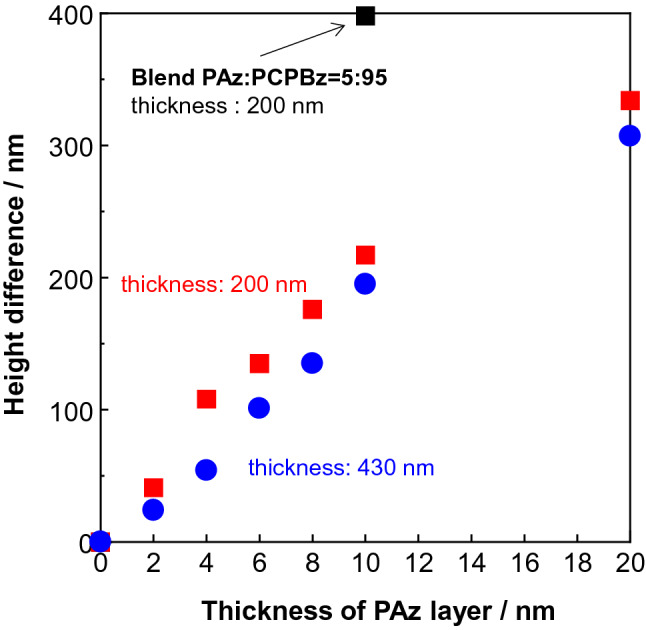
Top-to-bottom height difference of the SRG structure for PCPBz films with various deposition numbers of the PAz LS layer (layer thickness). Red and blue correspond to data for PCPBz films with initial thicknesses of 200 and 430 nm, respectively. Black square indicates data obtained for a 200-nm-thick surface segregated blended film (PAz:PCPBz = 5:95) film.

Figure 4 shows the top-to-bottom height difference as a function of the LS layer thickness of PAz. As the PAz layer thickness increased, the height difference was enhanced almost linearly. The PAz skin layer triggered mass motion of PCPBz in an amplified manner. Roughly 20 times larger thickness of PCPBz is affected from the PAz surface layer at each thickness. Our previous inkjet-printing study^40^ revealed that a larger amount of surface ink results in a larger flow motion. The observed thickness dependence herein could be explained in the same manner. As the PCPBz base film thickness increased to 430 nm (blue symbols), the protrusion-trench height difference systematically became smaller compared to the case with a 200-nm thickness. It seems that the convection flow is more favourable for a thicker film, allowing back flow in the bottom region.

A SCLCP with a cyanobiphenyl mesogen (PCB)^42^ was covered with a 5-layered or 10-layered PAz LS film and photoirradiated using the same procedure (Fig. S7). A smectic A phase appeared at 90 °C. In this case, mass transport only appeared in the surface layer of PAz, and the base film of PCB was not transported. This drastically different behaviour is attributed to the viscosity difference between the nematic (PCPBz) and smectic A (PCB) phases. For SCLCPs, an appreciable viscosity change between the nematic and isotropic thermal transition was not observed^55^. In contrast, the viscosity discontinuously changed with different orders of magnitude between the smectic and isotropic phases^40,55^. Our previous SRG systems of SCLCPs demonstrated that mass transport occurs from the trans-Az side (smectic A) area toward the cis-Az rich (isotropic) one^21–26^. Hence, a photoinduced phase transition to the isotropic state is necessary to reduce the film viscosity, and the previously observed SRG generation^21–26^ seems to be due to the Marangoni flow.

### PCPBz films with a surface segregated PAz layer

The surface segregation procedure is simple and very practical to set a photoresponsive skin layer. A 200-nm-thick mixed film of PAz and PCPBz (5:95 by weight) was annealed at 90 °C for 10 min. The *θ*_gly_ values of a mixture of PAz and PCPBz (5:95 by weight) after annealing at 90 °C for 10 min before and after UV irradiation were 99.2 ± 1.6° and 89.4 ± 1.6° at 25 °C, which agree well with those of the pure PAz film (Table 1). These results indicate that annealing selectively segregates the PAz component to the free surface^41–43^.

UV light irradiation was performed onto this surface segregated film through a photomask (stripe pitch: 20 µm) for 300 s at 90 °C. Photoirradiation efficiently generated the SRG corresponding to the photomask pattern (Fig. S8). The pattern formation was more efficient than that of the LS transferred one. The surface segregated skin layer of PAz should correspond to that of 10-nm-thick 5-layered LS film. The black square in Fig. 4 indicates the height difference obtained in this procedure. The significantly larger transport motion is attributed to the stronger molecular interactions between the top skin layer and the inner mesogens. The surface segregated PAz should have more significant interactions between the different mesogens with the concentration gradient at the boundary region of the two polymers. In contrast, the films were separately prepared and physically transferred in the LS deposition system, resulting in inefficient mesogen interactions between the layers.

### PAz films with a PC18 LS layer

Next, the system of a photoresponsive film covered with a photo-inert surface was explored (Fig. 1c). A photo-inert long-chain side-chain polymer (PC18) layer was transferred by the LS method onto a PAz film, Then UV light was irradiated through a photomask. PC18 monolayer deposition was carried out under the same conditions used to form a monolayer spreading film of PAz on water (Fig. S4). The PC18 layer was selected because LS deposition is readily performed and does not absorb light in the UV wavelength region. Expectedly, the PC18 molecular layer inhibited the surface tension difference of the inner PAz film due to UV light irradiation.

**Figure 5:**
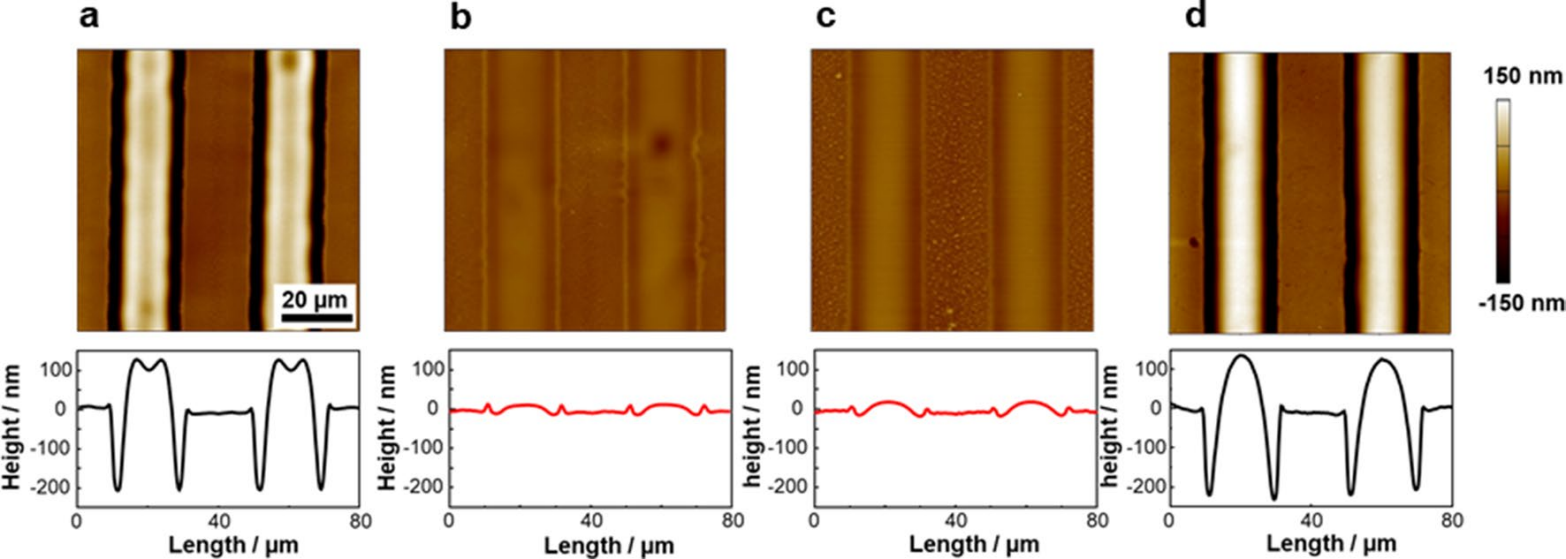
Suppression of mass transport of a 200-nm-thick PAz film by a PC18 top layer. Topographical AFM images (upper) after cross-sectional height profile (lower) stripe-patterned UV irradiation at 90 °C on a pure PAz film (**a**) and those with a 1-layered (**b**) and 3-layered (**c**) PC18 LS films on the surface. In (**d**), the same data is shown after removal of the PAz LS monolayer.

**Figure 6:**
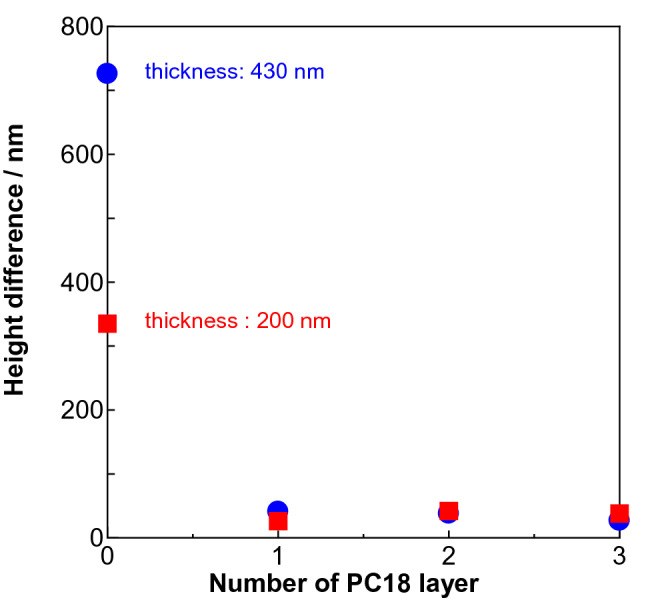
Top-to-bottom height difference of the SRG structure of PAz films with various deposition numbers of the PC18 LS layers. Patterned irradiation was performed at 90 °C. Red and blue correspond to data for PCPBz films with initial thicknesses of 200 and 430 nm, respectively.

As a control experiment, a pure 200-nm-thick PAz film was first exposed to patterned UV irradiation (conventional system, corresponding to Fig. 1a). As previously confirmed^25^, the SRG structure was generated efficiently and the top-to-bottom height reached to 335 nm (Fig. 5a). When a PC18 monolayer was transferred to the PAz film by the LS method, the transport motion was greatly hindered, and the height difference was only 30 nm (Fig. 5b). Therefore, the PC18 monolayer approximately 1–2-nm thick^56^ strongly suppressed the formation of SRG, although more than 99% of the film component was composed of PAz. The surface of the PAz film covered with PC18 monolayer gave *θ*_gly_ of 106.9 ± 0.6° and 106.8 ± 0.6° before and after UV irradiation, respectively, at 25 °C. These results agree precisely with the pure PC18 film surface without changes upon UV light irradiation. Thus, one monolayer can prevent a photoinduced surface tension change. Further deposition of three layers of PC18 did not enhance the effect (Fig. 5c). Figure 6 shows the above situation. To prevent mass transfer motion, one monolayer coverage is sufficient regardless of the initial film thickness. The minor motions leading to 30 nm undulations may be attributed to the effect of the large viscosity alternation formed at the boundary between the smectic A and isotropic phases^40^. In this UV irradiation condition, photoisomerisation to the cis-rich photostationary state fully proceeded. To confirm the role of the surface, the PC18 monolayer was successively removed by rinsing the film in cyclohexane at 20 °C (Figs. S9 and S10). When the monolayer was detached, the large transport motion was recovered (Fig. 5d).

Viswanathan et al.^57^ demonstrated that the deposition of layer-by-layer polyelectrolyte ultrathin film on an amorphous Az polymer significantly impeded the mass transport motions. Later, Ober’s group^58^ showed that films of Az SCLCPs with semifluorinated tails at the Az mesogen displayed a similar restriction. However, these investigations did not mention the surface tension change upon irradiation. Since the hinderance of the migration motion is similar with our results and our PC18 monolayer hardly has a steric constraint, the contribution of the surface tension changes must be considered, even for the former studies^57^^,^^58^.

### SRG film essentially without colour

The SRG systems based on Az polymers are coloured in yellow or red. The Az unit is essential for the photoinduced mass migration, but the existence of this strongly light absorbing chromophore can be a drawback after relief formation for many optical applications such as optical storage, waveguide couplers, liquid crystal alignment layers, etc. Therefore, SRG systems without colour are favoured. So far, post-decolouration of Az polymer films have been attempted by detaching the Az unit via solvent extraction from supramolecular polymers^24,59^ or by heat-cleavage of the chemical bond^60^.

**Figure 7:**
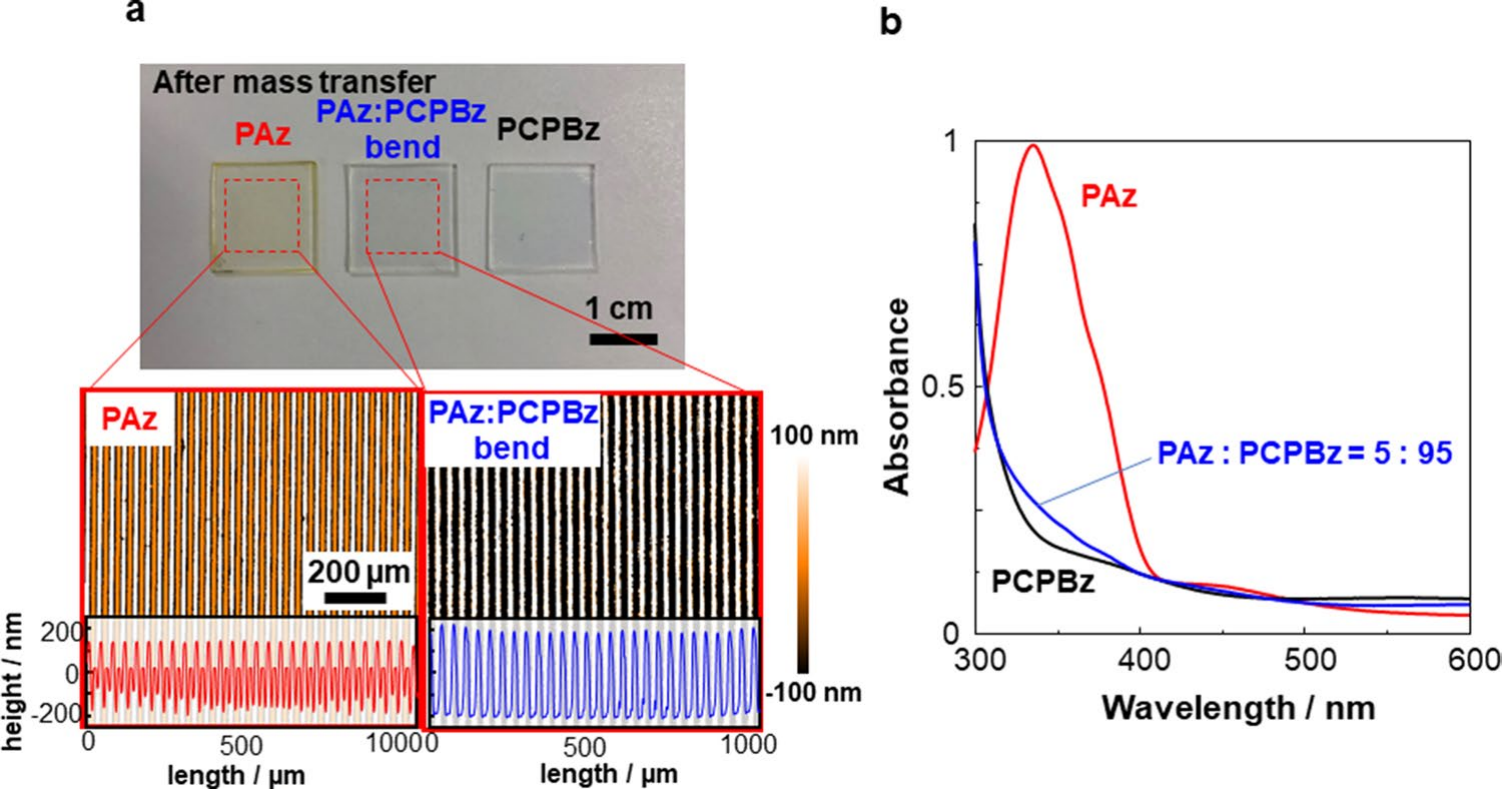
SRG films after patterned irradiation at 90 °C. Photos of SRG films of a pure PAz film (**a**, left), surface segregated blended (PAz:PCPBz (5:95)) film (**a**, middle), and pure PCPBz film (**a**, right). UV–visible absorption spectra before light irradiation (**b**). All samples are 200-nm thick.

This study offers another strategy to provide dim coloured SRG films. Figure 7 displays photos of pure PAz, PAz-PCPBz blend (5:95) and pure PCPBz films after irradiation with patterned UV light. The pure PAz (left) and PCPBz (right) films were yellowish and colourless as recognised by the naked eye, respectively. Similar to the PCPBz film (right), the surface segregated PAz/PCPBz film (middle) was almost colourless (Fig. 7a). The difference was obvious in the UV–visible absorption spectra. For the surface segregated blended film, the large π–π* absorption band peaking at 348 nm was significantly reduced in the blend film, generating a spectrum similar to that of pure PCPBz film. In this way, the selective introduction of Az unit at the surface can be a facile and useful strategy to fabricate essentially colourless SRG films. Expectedly, selective removal of the top PAz layer by rinsing with an appropriate solvent as proven in Figs. S9 and S10 and previously^42^ can provide a fully colourless SRG film.

### Revisiting previous SRG studies

Although the suppression of SRG formation by the surface layer has been reported^57,58^, this work demonstrates, for the first time, photoinduction of the surface morphology for photo-inert polymer films by the photoactive surface layer. To date, SRG studies have been conducted for films with photoreactive units existing in the entire film. For these films, photoirradiation induces multiple changes simultaneously such as a viscosity change, molecular order and orientation change, and surface tension change. Kim et al.^36^ showed the surface fabrication of an Az polymer via the Marangoni flow, but these other factors may also be involved.

Our strategy of preparing a surface skin layer provides a system that allows arguments purely based on the surface effect. It is confirmed that the surface tension gradient should be the primary factor, and the other physical changes inside the film are negligible in our system. A small change in the surface tension can cause large mass convection in polymer films^39^. Hence, the surface tension gradient should be taken into more consideration in SRG studies. For example, Koskela et al.^28^ reported interesting data where only 1–2% introduction of an Az chromophore into a polymer via a supramolecular framework can induce SRG structures. It is possible that the surface segregation of more hydrophobic Az molecules to the surface facilitates the mass transport process.

In our SCLCP films, the transport direction was independent of linear light polarisation (Fig. S6)^54^, strongly suggesting that the Marangoni effect is the plausible mechanism. In most of SRG studies using amorphous Az polymers, a strong polarisation dependence has been observed. Such a polarisation dependence cannot be explained by the surface tension gradient. Angular selective excitation and orientation effects are involved in such systems. Hence, complex factors should be involved to precisely understand morphology induction. Very recently, Miniewicz et al.^61^ argued the surface morphing behaviour of an Az polymer film by changing the laser beam intensities. Based on their observations and modelling, the significant role of the Marangoni effect is also emphasized. Frascella et al.^62^ recently reported that the SRG formation is not much affected by covering a photo-inert layer for an ionic amorphous azobenzene polymer system, which is seemingly contradictory to our results. The discrepancy should stem from the difference in the driving mechanism. The polyelectrolyte system by Frascella et al. is amorphous and the migration direction is highly dependent on the polarisation. In such system the motion is possibly driven by gradient force^9–11^ and/or molecular orientation force^19,20^. In contrast, in Az-containing SCLCP films, the motion is driven thermally by the instability of the surface tension gradient as demonstrated in this paper. Such difference in the mechanism can be understood by a large difference in the light intensity required. The experiments by Frascella et al. are conducted at 250 mW cm^–2^, and in our system light intensity at 1.0 mW cm^–2^ is sufficient.

## Conclusion

This study demonstrated the essential contribution of the topmost surface to the macroscopic mass transport process in SCLCP films. The presence of only a molecular level skin layer at the free surface is sufficient to promote or terminate a large-scale surface deformation. These results are well understood by the Marangoni flow driven by the light-triggered surface tension instability. Although only a few previous reports mentioned the significance of the surface effect^57,58^, several recent studies emphasised the importance of surface proximities for both LC^40,58^ and amorphous^17,18,36,57^ polymers. To precisely understand the behaviour, the driving mechanism, including the surface effect, should be reconsidered.

In the surface photoalignment of LC systems, a small amount of photoreactive molecules on a solid substrate can control the alignment of a large number of non-photoresponsive LC molecules. Such a surface is called the command layer^45,52,63^. Recent investigations have revealed that a photoactive command layer can be placed at the free surface in SCLCP films^41–47^. Similarly, the present work offers another command layer scenario where large molecular amplification is reflected in macroscopic surface morphing. This strategy should realise light-assisted microfabrications for light-inert polymer films.

## Experimental methods

### Polymers

Details of the polymer syntheses, their characterisations, and experimental procedures are described in the Supplementary Information.

### Preparation of films

Polymer ultrathin films with molecularly controlled thicknesses were prepared by the Langmuir-Schaefer (LS) method. PCPBz films (base films) were prepared by spin coating (Mikasa, Japan) from a 3.0 or 4.5% chloroform solution by weight onto a quartz substrate, yielding typical film thicknesses of approximately 200 and 430 nm, respectively. The PAz solution (ca. 1.0 × 10^–3^ mol dm^–3^ per Az monomer unit) in chloroform was prepared. The spreading behaviour of the PAz monolayer was characterised by a Lauda film balance (FW-1, Germany) filled with pure water at 20 °C. The sliding barrier was compressed at a speed of 30 cm^2^ min^–1^. The floating PAz monolayer obtained on the water surface was transferred onto a PCPBz film by the LS method at a surface pressure of 15 mN m^–1^. The same procedure was used to transfer the PC18 monolayer on water onto the PAz film.

Surface segregated films of PAz layer/PCPBz were prepared from a 3% mixed solution of chloroform containing 5% of PAz to PCPBz by weight. The films were subsequently annealed at 90 °C for 10 min.

### Methods

The film thicknesses were evaluated with white interferometric microscopy (WLIM, BE-S501, Nikon, Japan). Static water contact angles of water (*θ*_w_, surface tension, *γ*_w_ = 72 mN m^–1^ at 25 °C) and glycerol (*θ*_gly_, *γ*_gly_ = 63 mN m^–1^ at 25 °C) droplets on these polymer films were obtained by a contact angle meter (CA-XP, Kyowa Interface Science, Japan). The contact angle values were the average of at least five repeated measurements. Glycerol was used for the requirement of measurements at high temperatures such as 90 °C. UV-light irradiation (365 nm) was performed with a mercury-xenon lamp (Sanei Electronics Supercure 203S, Japan) at 1.0 mW cm^–2^ (365 nm) at 90 °C. Patterned UV irradiation was achieved through a photomask, which typically had a 20-µm stripe pitch (Edmond Optics, USA) (Fig. S11). The 365-nm line was selected by passing through combinations of glass filters of UV-35/UV35D (Toshiba, Japan). The UV–visible absorption spectra measurements were performed on an Agilent 8453 spectrometer (Agilent Technologies, USA). The morphological changes were observed by WLIM as mentioned above or via atomic force microscopy (AFM) (MFP-3D, Oxford Asylum Research, UK).

## Supplementary information

Supplementary file1
