# Supplementary material for: Photo-triggered large mass transport driven only by a photoresponsive surface skin layer
Source: Sci Rep. 2020 Jul 29;10:12664. doi: 10.1038/s41598-020-69605-8 (PMC7391747; doi:10.1038/s41598-020-69605-8)
Supplement: Supplementary file 1 — Supplementary file1 [file 41598_2020_69605_MOESM1_ESM.pdf]

Supplementary Information for

Photo-triggered large mass transport driven only by a photoresponsive surface skin layer

Issei Kitamura, Keisuke Kato, Rafael Benjamin Berk, Takashi Nakai, Mitsuo Hara, Shusaku Nagano, Takahiro Seki

Contents:

Polymer synthesis
Supplementary Figures S1 – S11.

Polymer Synthesis

1. Materials

Methyl -4-hydroxybenzoate, 6-bromo-1-hexanol, potassium carbonate, Triethylamine (Et_3N), 4-Hydroxybenzonitrile and 1-(3-Dimethylaminopropyl)-3-ethylcarbodiimide (EDC) were obtained from TCI. Unless stated otherwise, all reagents and solvents were used as received.

2. Synthesis of 4-cyanophenyl-4'-(6-acryloxyhexyloxy) benzoate (CPBz) monomers

4-Cyanophenyl-4'-(6-acryloxyhexyloxy) benzoate (CPBz) was synthesized as described in a previous paper[S1].

2-1. Synthesis of Methyl-4-(6-hydroxyhexyloxy) benzoate

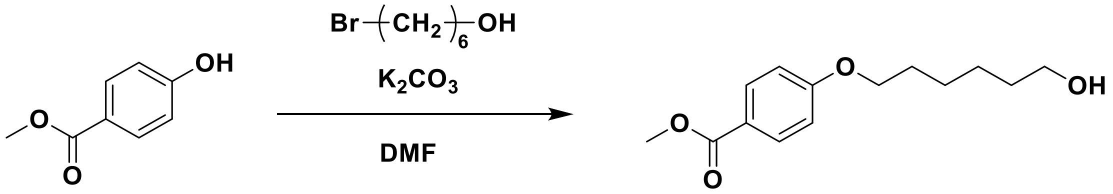

Potassium carbonate (28.6 g, 0.21 mol) was added to a dry *N,N*'-dimethylformamide (DMF) solution containing methyl-4-(6-hydroxyhexyloxy) (20.9 g, 0.14 mol) under N_2 gas. 6-Bromo-1-hexanol (22.6 mg, 0.17 mol) in dry DMF was then added dropwise under stirring. The solution was stirred for 8 h at 80 °C. After this procedure, the solution was dissolved in water, and extracted with ethyl acetate and water. The precipitate was recrystallized from ethyl acetate to give white powdery crystals. Yield: 30.5 g (87.6 %).

^{1}H-NMR (400 MHz, $CDCl_3$): δ (ppm) = 1.36-1.44 (6H, m, -C**H**$_2$-), 1.71-1.75 (2H, m, -C**H**$_2$-), 3.75 (3H, s, -O-C**H**$_3$), 4.01-4.05 (2H, t, -C**H**$_2$-OH), 4.35-4.38 (2H, q, -O-C**H**$_2$-), 6.99-7.01 (2H, d, Ar-**H**), 7.86-7.89 (2H, d, Ar-**H**)

2-2. Synthesis of 4-(6-hydroxyhexyloxy) benzoic acid

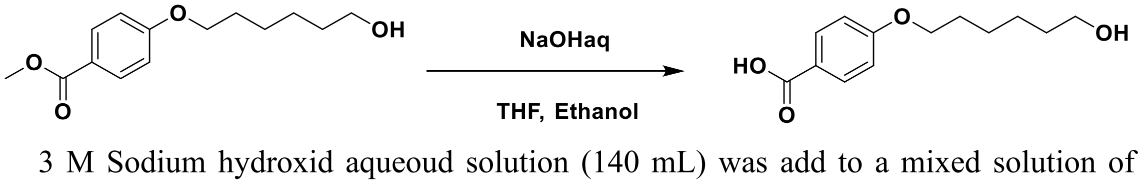

3 M Sodium hydroxid aqueoud solution (140 mL) was add to a mixed solution of

purified tetrahydrofuran (THF) and ethanol containing 4-(6-hydroxyhexyloxy)benzoic acid (30.5 g, 0.12 mol). The solution was stirred for 24 h at 25 °C. After this procedure, the solution was dissolved in water, and extracted with THF and water. The precipitate was recrystallized from THF to give white powdery crystals. Yield: 23.1 g (70.2 %).

^{1}H-NMR (400 MHz, $CDCl_3$): δ (ppm) = 1.36-1.44 (6H, m, -C**H**$_2$-), 1.71-1.75 (2H, m, -C**H**$_2$-), 4.01-4.05 (2H, t, -C**H**$_2$-OH), 4.35-4.38 (2H, q, -O-C**H**$_2$-), 6.99-7.01 (2H, d, Ar-**H**), 7.86-7.89 (2H, d, Ar-**H**), 12.6 (-C(=O)O-**H**)

2-3. Synthesis of 4-(6-acryloxyhexyloxy) benzoic acid

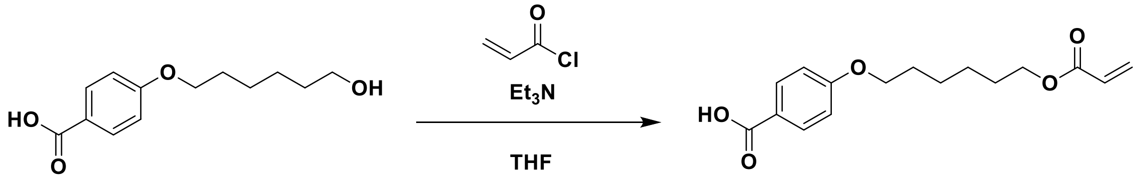

Triethylamine (26.9 mL, 0.19 mol) was added to a purified THF solution containing 4-(6-hydroxyhexyloxy) benzoic acid (23.1 g, 0.097 mol) under N_2 gas. A purified THF solution of acryloyl chloride (11.7 mL, 0.15 mol) was added dropwise to the solution at 5 ºC. The mixture was stirred for 24 h at 25 °C. After this procedure, the solution was dissolved in chloroform and water, and extracted with ethyl acetate and water. The precipitate was recrystallized from ethyl acetate and hexane to give white powdery crystals. Yield: 19.7 g (69.5 %).

^{1}H-NMR (400 MHz, $CDCl_3$): δ (ppm) = 1.41 (6H, m, -C**H**$_2$-), 1.62-1.74 (2H, m, -C**H**$_2$-), 4.02-4.05 (2H, t, -C**H**$_2$-O-C(=O)-), 4.09-4.13 (2H, q, -O-C**H**$_2$-), 5.92-5.95, 6.29-6.34 (CH=C**H**$_2$), 6.14-6.20 (C**H**=CH$_2$), 6.98-7.01 (2H, d, Ar-**H**), 7.86-7.88 (2H, d, Ar-**H**), 12.5 (-C(=O)O-**H**)

2-4. Synthesis of 4-cyanophenyl-4'-(6-acryloxyhexyloxy) benzoate (CPBz)

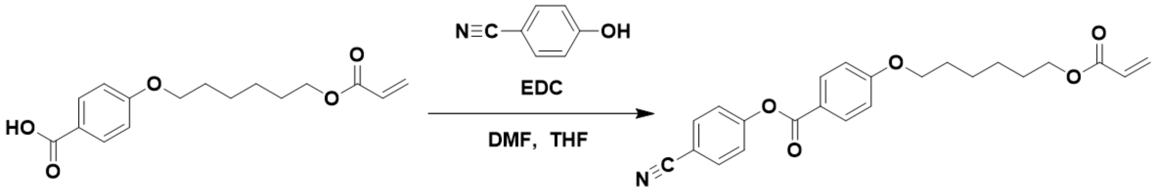

1-(3-Dimethylaminopropyl)-3-ethylcarbodiimide (EDC, 24.0 mL, 0.14 mol) in purified THF was added dropwise to a mixed solution of THF and DMF containing 4-(6-acryloxyhexyloxy) benzoic acid and 4-hydroxybenzonitrile under N_2 gas. The mixture was stirred for 7 days at 25 °C. After this procedure, the solution was dissolved in chloroform and water, and the crude product was extracted with chloroform and water. The precipitate was recrystallized from ethyl acetate to give white powdery

crystals. Yield: 11.5 g (42.9 %).

^{1}H-NMR (400 MHz, $CDCl_3$): δ (ppm) = 1.47-1.87 (8H, m, -C**H**$_2$-), 4.04-4.08 (2H, t, -C**H**$_2$-O-C(=O)-), 4.17-4.20 (2H, q, -O-C**H**$_2$-), 5.81-5.84, 6.39-6.43 (CH=C**H**$_2$), 6.09-6.16 (C**H**=CH$_2$), 6.97-6.99 (2H, d, Ar-**H**), 7.26-7.37 (2H, d, Ar-**H**), 7.73-7.75 (2H, d, Ar-**H**), 8.12-8.14 (2H, d, Ar-**H**)

2.5 Polymerization of CPBz via atom transfer radical polymerization

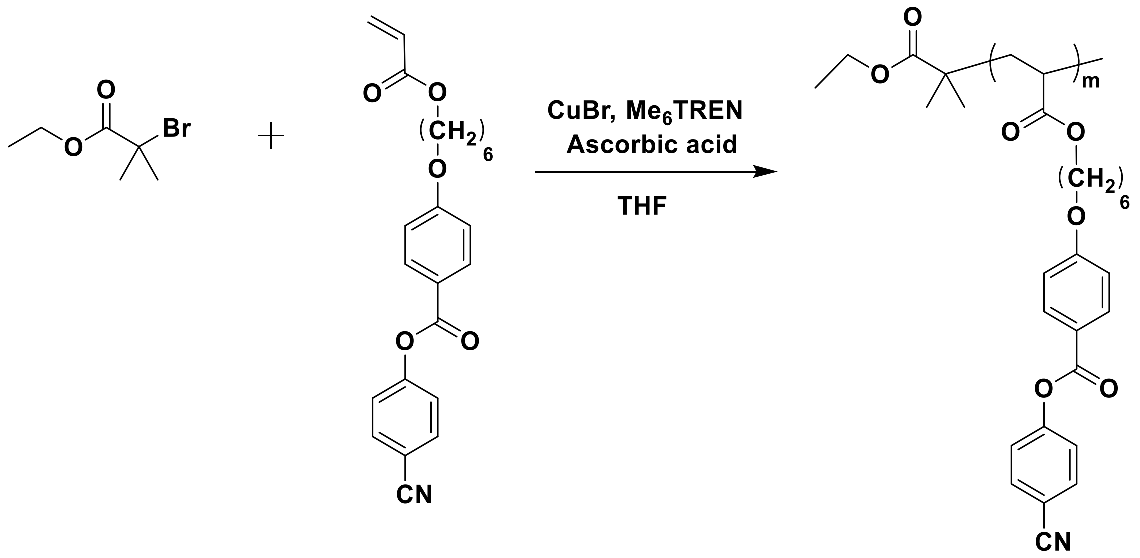

Polymerization of PCPBz was achieved by the atom transfer radical polymerization (ATRP) method.

Cu(I)Br (1.5 mg, 0.01 mmol), Me_6TREN (3.3 mg, 0.01 mmol), ascorbic acid (9.0mg, 0.05 mmol) and CPBz monomer 1000 mg, 2.5 mmol) were put into a 15 mL pressure tube and purged with a N_2 gas. In a glove box, dry THF (1.32 mL, 2mol dm^{-3} for CPBz monomer) was added to the tube and stirred for 5 min. Polymerization started by injection of a initiator, ethyl-2-bromoisobutyrate (EBB) (1.3 μL, 0.01 mmol), and the sealed tube was moved from the glove box to a personal synthesizer (ChemiStation, EYELA, Japan) and stirred for 24 h at 70 °C. The polymerization was quenched by exposure the reaction solution to air. The solution was pass through an activated neutral alumina column to remove the Cu catalyst with chloroform as the eluent. The solution was concentrated by evaporation, and the product was precipitated by pouring into methanol.

The average number of repeating unit of Az was 129 ($PCPBz_{129}$); M_n= 5.1× 10^4 (^{1}H-NMR), M_w/M_n = 1.19 (GPC). Thermophysical properties evaluated by DSC: glass-23 °C - nematic - 114 °C - isotropic.

2.6 Polymerization of PAz via atom transfer radical polymerization

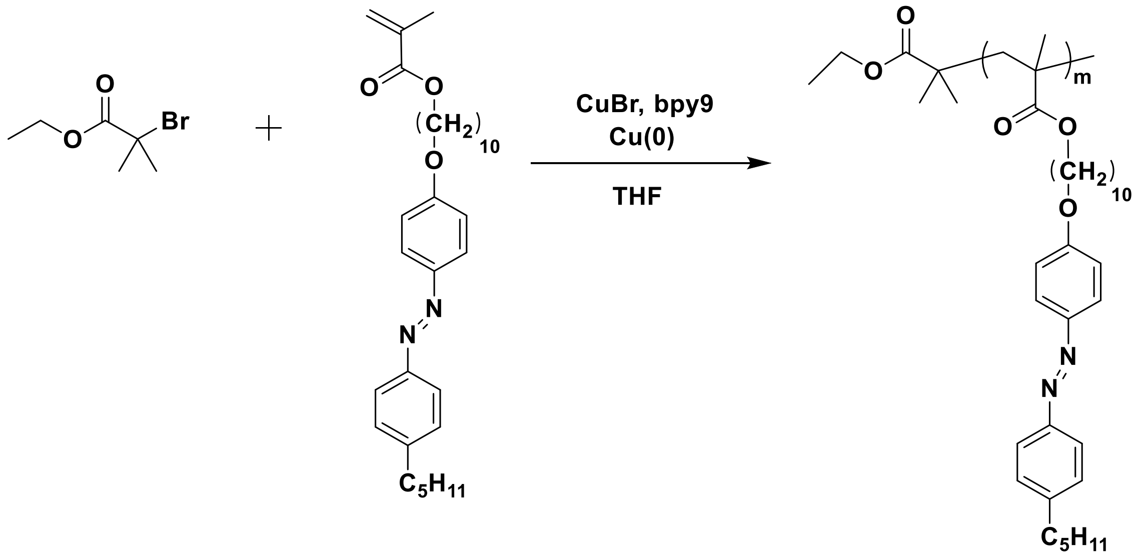

PAz was synthesized by the ATRP method according to the previous paper[S2]. The average number of repeating unit of Az was 26 (PAz_{26}); M_n= 1.3×10^4 (NMR), M_w/M_n = 1.08 (GPC). Thermophysical properties evaluated by DSC: glass-45 °C - smectic C - 87 °C-smectic A –110 °C - isotropic.

References

S1) Noël, C., Friedrich, C., Leonard, V., Barny, L. P., Ravaux, G. & Dubois, J. C. Synthesis and characterization of side-chain liquid crystalline copolymers for non-linear optics. *Makromol. Chem. Macromol. Symp.* **24**(1), 283-301 (1989).

S2) Nakai, T., Tanaka, D., Hara, M., Nagano, S. & Seki, T. Free surface command layer for photoswitchable out-of-plane alignment control in liquid crystalline polymer films. *Langmuir* **32**, 909-914 (2016).

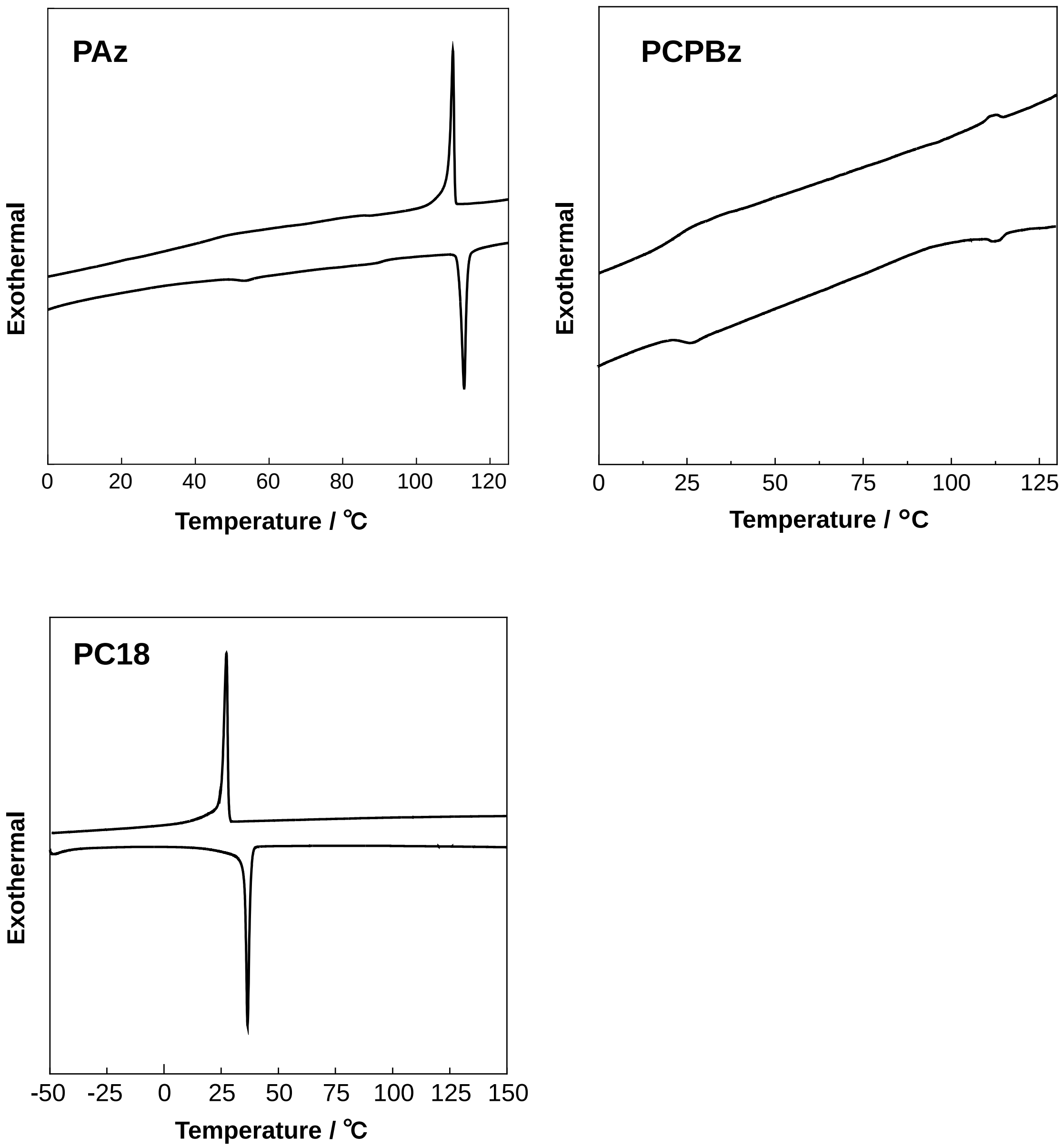

Figure S1. DSC curves of PAz, PCPBz and PC18. The phase transition temperatures are listed in Table 1 in the text.

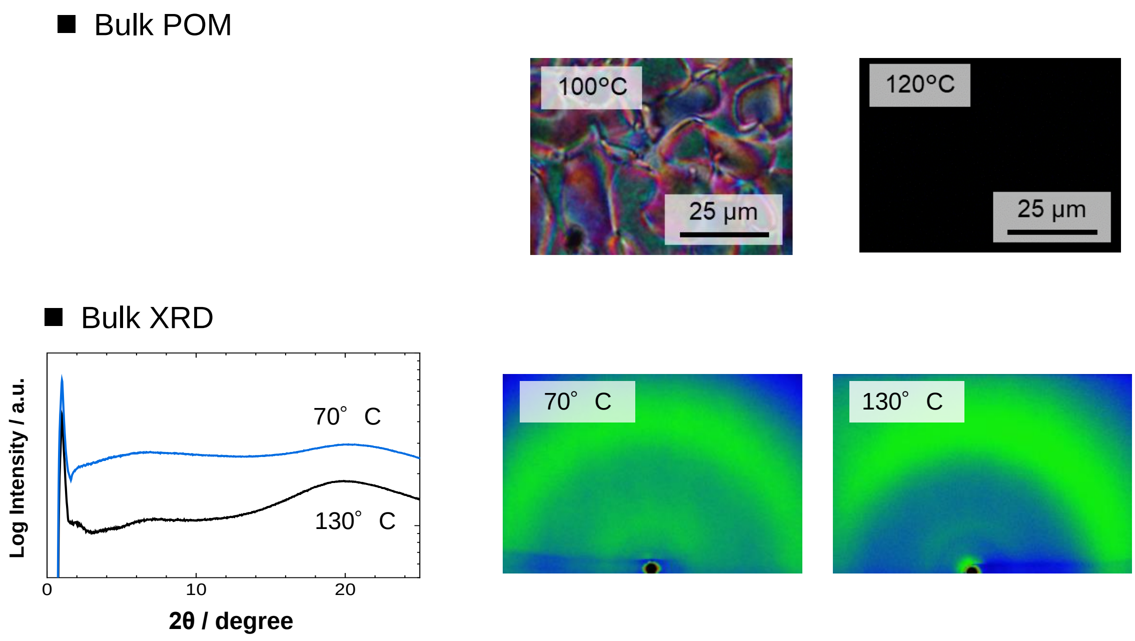

Figure S2. Characterisations of PCPBz. POM observations and X-ray scattering profiles. Schlieren texture at 100 °C in POM image and absence of peaks in small angle regions of X-ray scattering profiles indicate that this polymer exhibits a nematic phase.

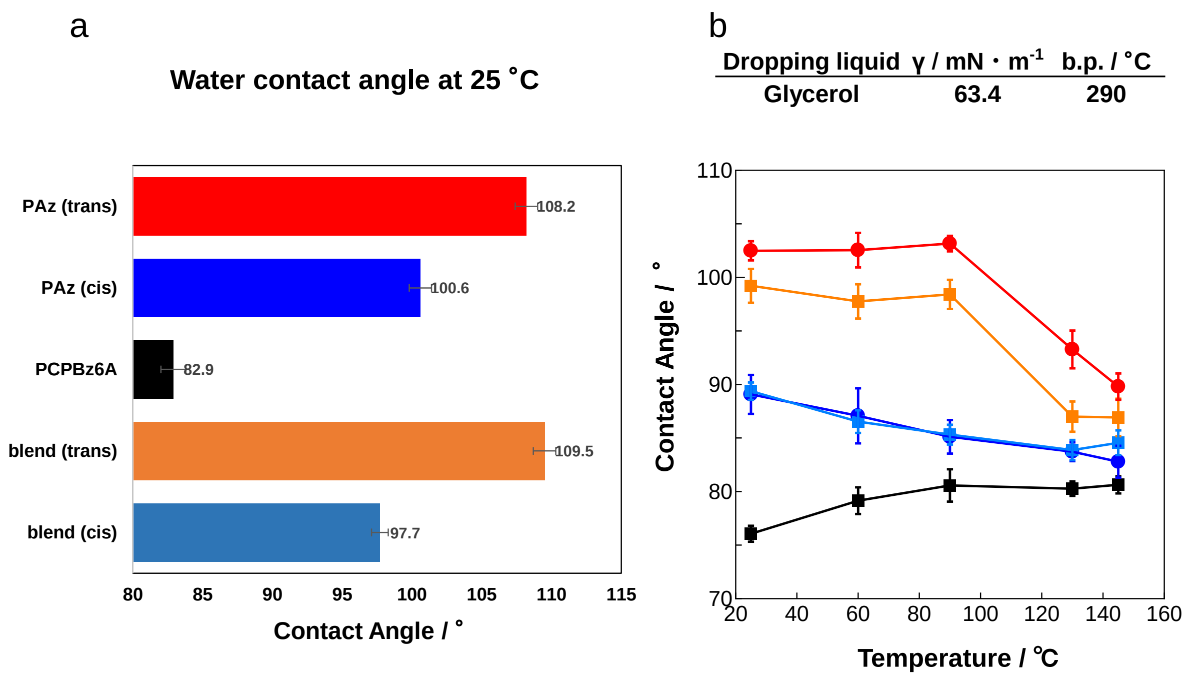

Figure S3. Contact angle data. (a) θ_{water} at 25 °C, and (b) θ_{gly} at various temperatures ranging 20 – 150 °C.

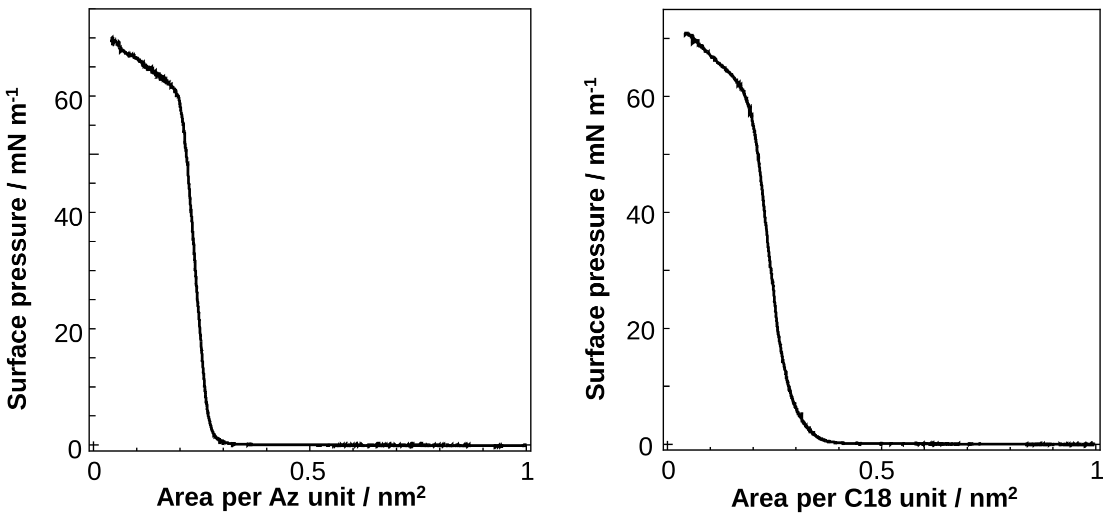

Figure S4. π-A isotherms of PAz (left) and PC18 (right) on pure water at 20 °C.

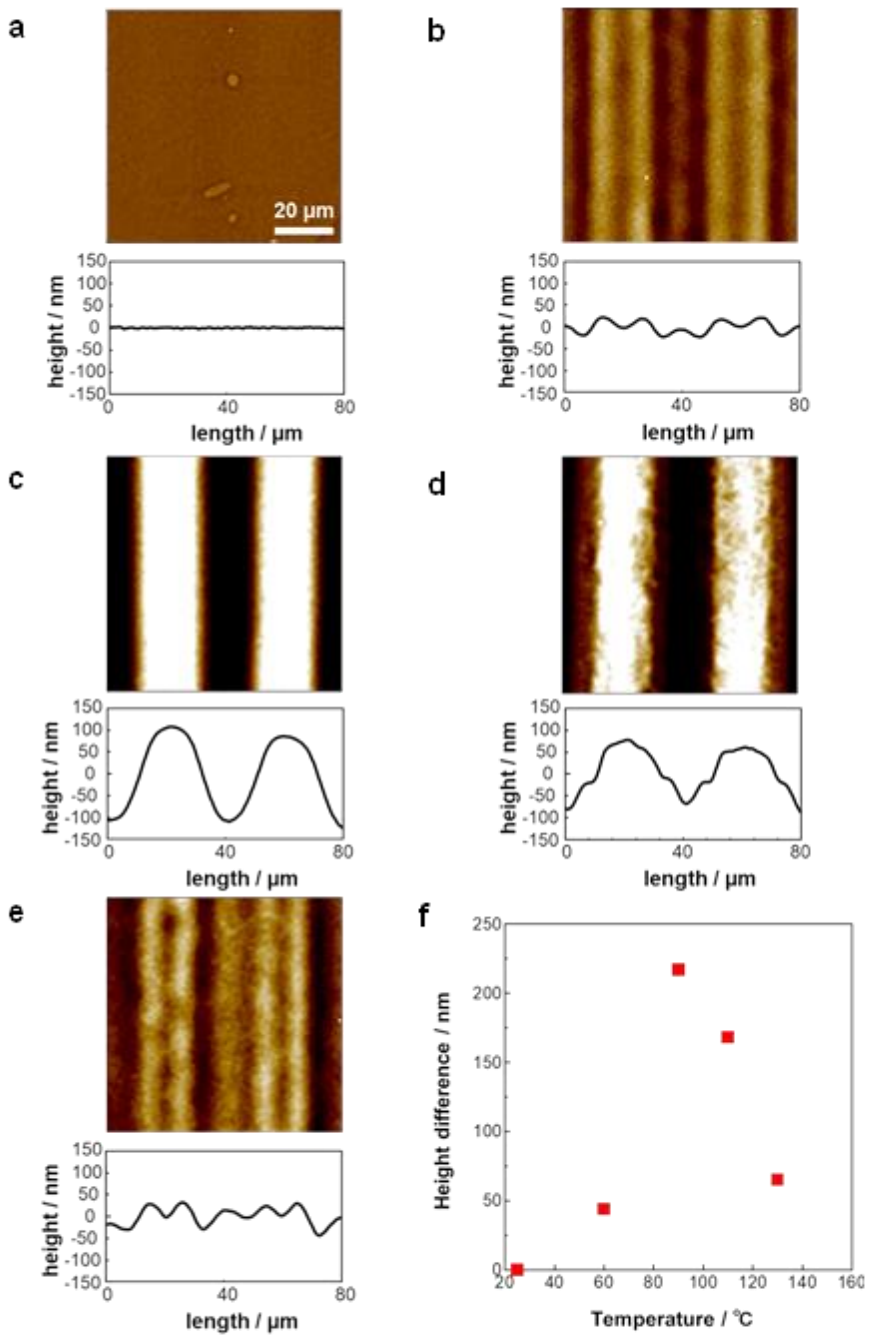

Figure S5. Photoinduced mass transport of 5-layerd PAz film on PCPBz film. UV irradiation is performed through a stripe photomask (20 μm pitch) at 25 °C (a), 60 °C (b), 90 °C (c), 110 °C (d), 130 °C (e). Top-to-bottom height difference of the SRG structure for 5-layerd PAz LS film on PCPBz films with various UV irradiation temperature (f).

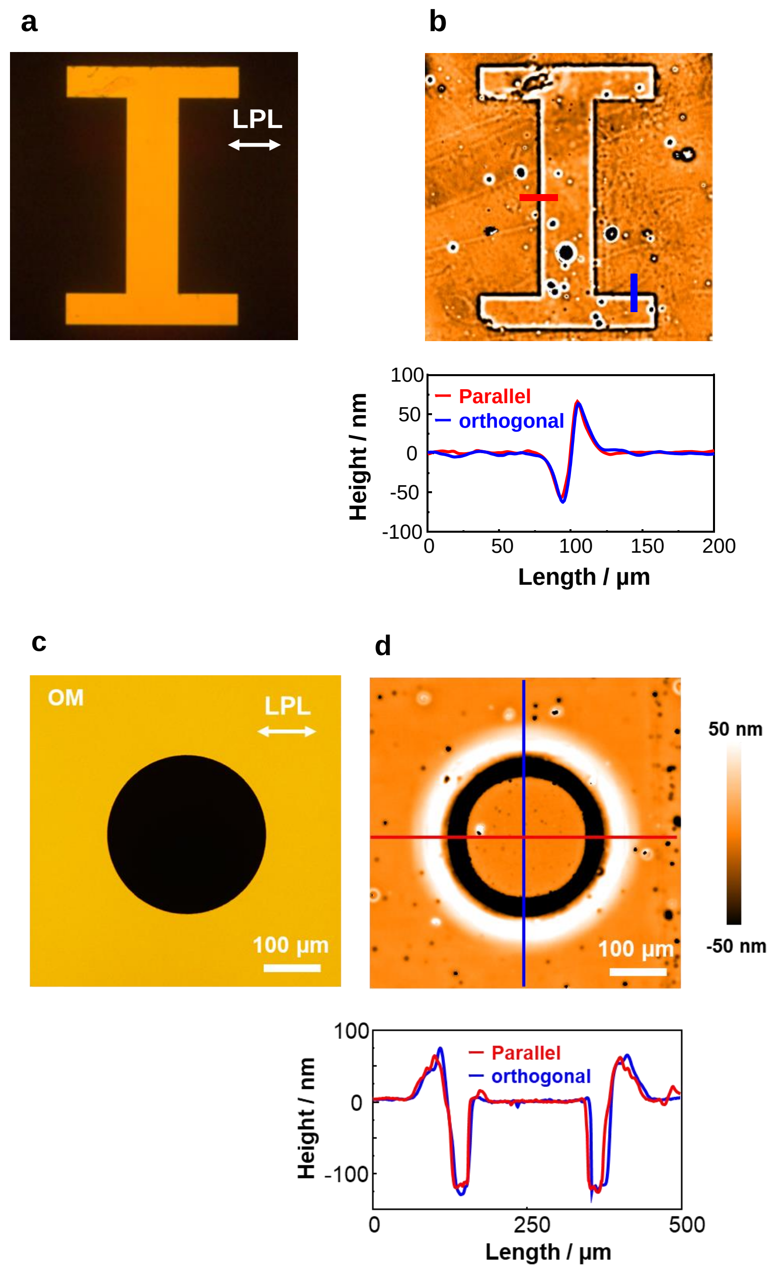

Figure S6. Polarisation dependence. The surface morphology of PCPBz film (200 nm) covered with a 5-layered PAz LS film (10 nm) after linearly polarised UV light for 100 s at 90 °C. Photomask (a) and resulting relief structure (b). The surface morphology of a pure PAz film (150 nm) after linearly polarized UV light for 300 s at 90 °C. Photomask (c) and resulting relief structure (d). In both systems, the surface profiles taken in parallel (red trace in b and d) and orthogonal (blue trace in b and d) to the linearly polarised UV light gave essentially the same surface profiles, indicating that no polarisation effect is involved in the transport process.

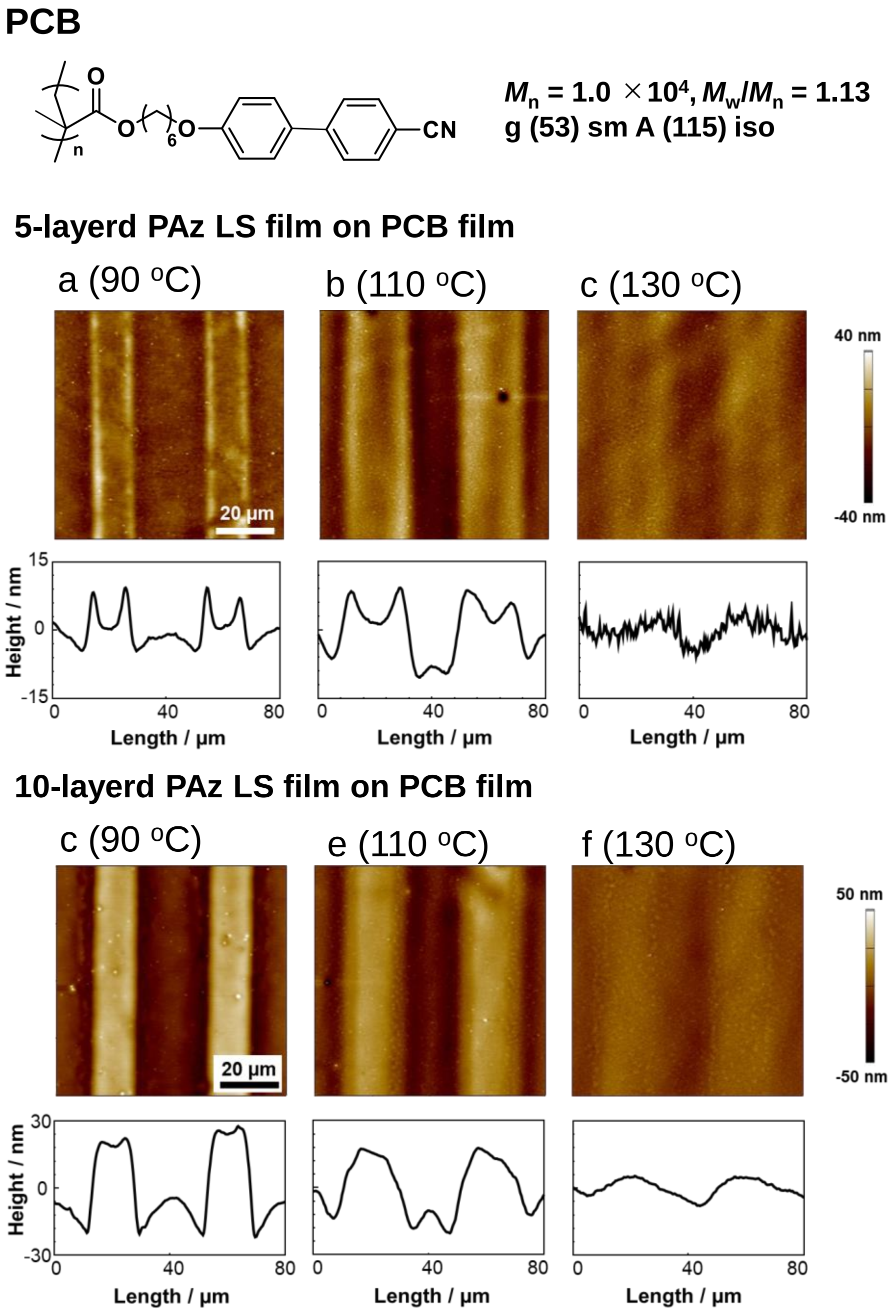

Figure S7. Photoinduced mass transport of PAz LS layer on PCB film. UV irradiation is performed through a stripe photomask (20 μm pitch). The layer numbers of PAz are 5 (a - c) and 10 (d - f). Note that only the surface PAz LS layer is transported as revealed from the top-to-bottom height, and the PCB film is not affected. Note that the height range is one-order smaller than that shown in Figures 3 or 5 in the main text. At 130 °C, the PCB film adopts a fluid isotropic state, however, the thermal back reaction of cis-Az unit becomes very fast, resulting in the less efficient surface relief formation.

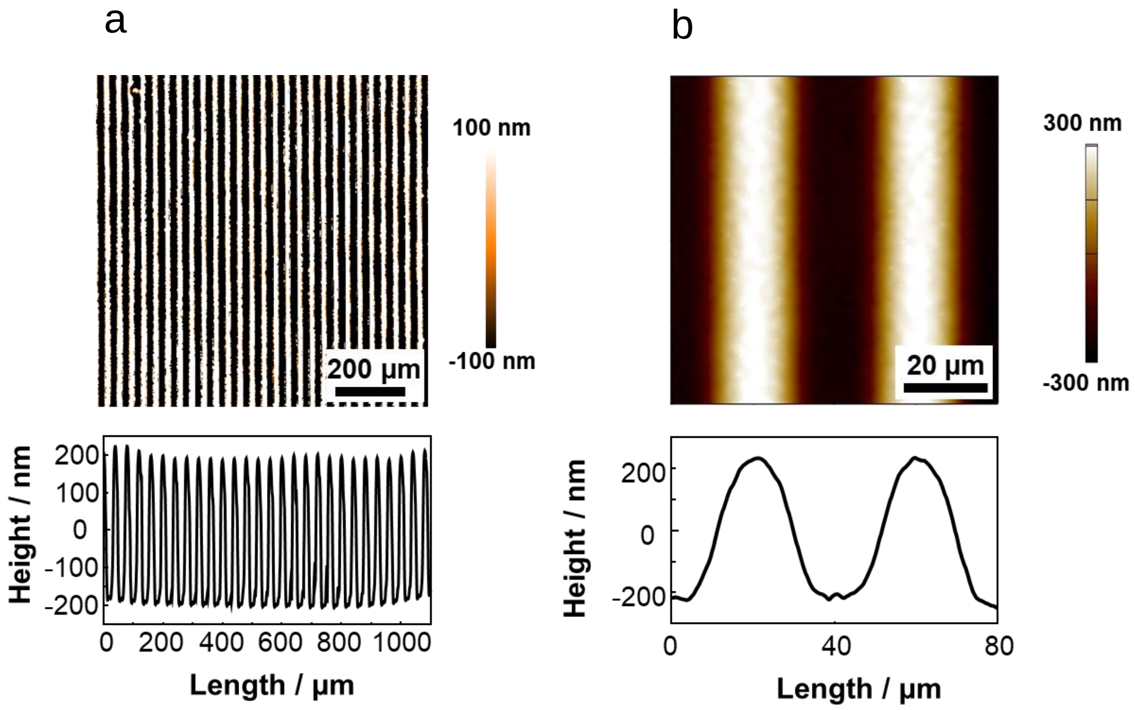

Figure S8. Photoinduced mass transport of surface segregated blended (PAz:PCPBz (5:95)) film. Topographical WLIM images (a) and AFM images (b) after UV irradiation at 90 °C through a stripe photomask (20 µm pitch).

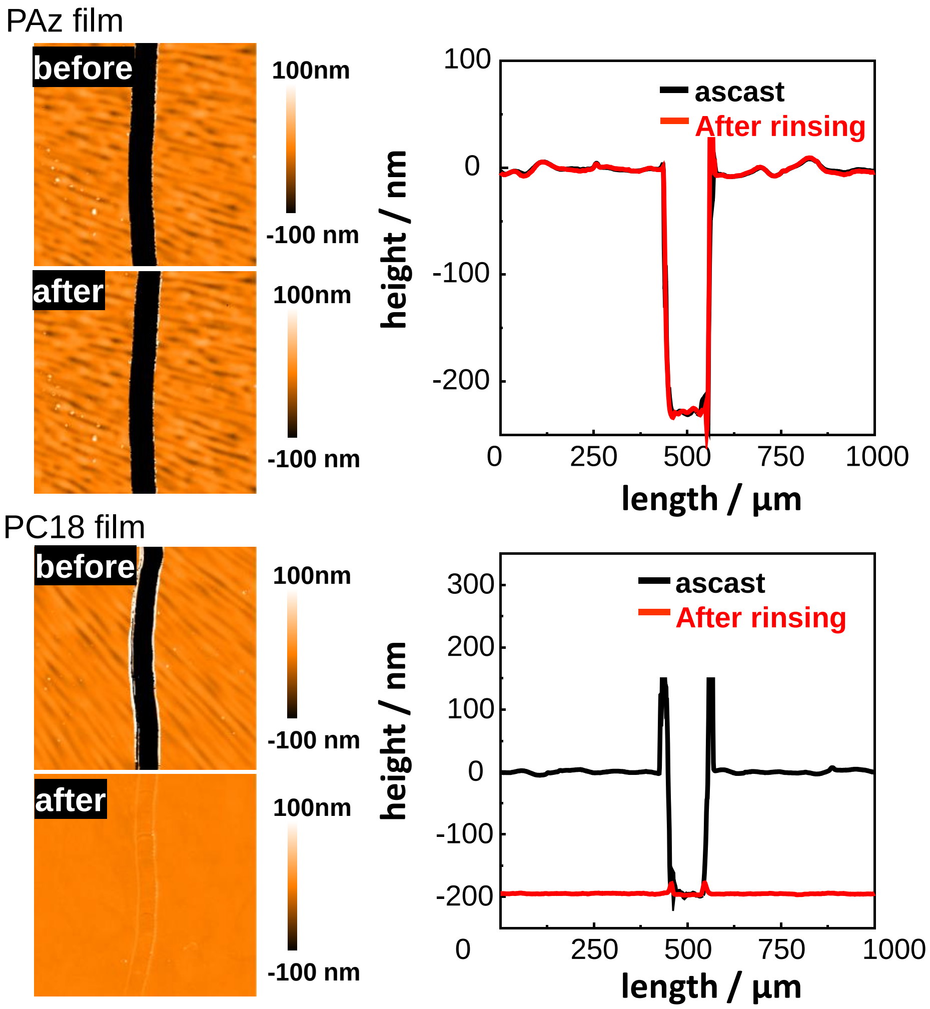

Figure S9. Solubility tests. Spincast films of pure PAz and PC18 were prepared (200 nm thickness), and then immersed in cyclohexane at 20 °C with shaking. Note that PAz does not dissolve in cyclohexane, and PC18 is fully dissolved in the same conditions.

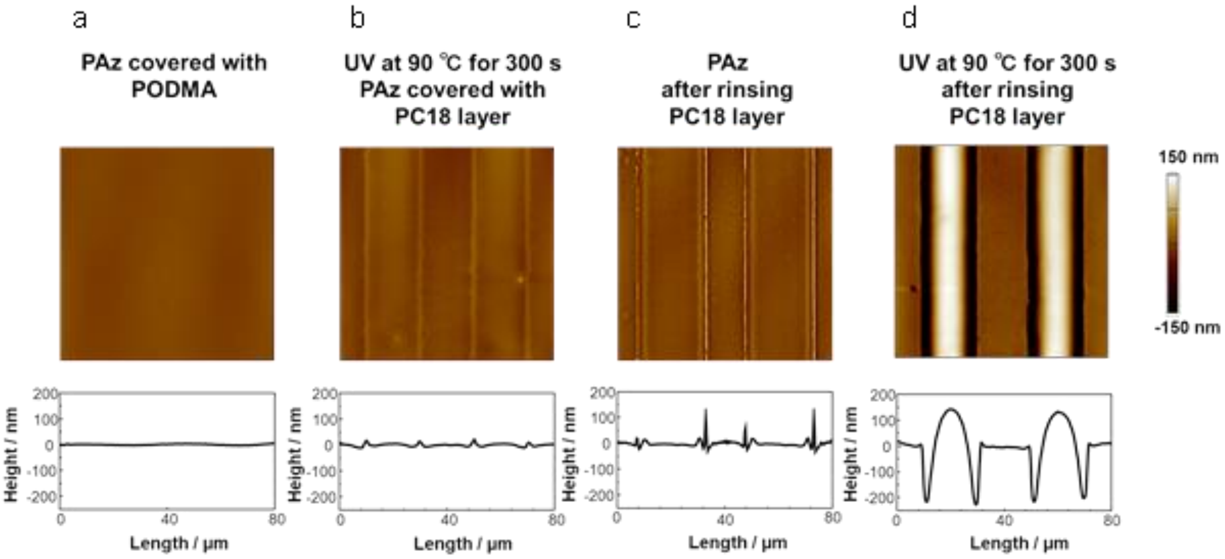

Figure S10. Topographical AFM images of a PAz film covered with PC18 LS monolayer. Initial flat surface (a), after UV irradiation through a line and space (20 µm pitch) photomask (b), after rinsing with cyclohexane (c), and patterned UV light irradiation of the rinsed film. Note that the induction of SRG formation is fully recovered after removal of PC18 LS monolayer.

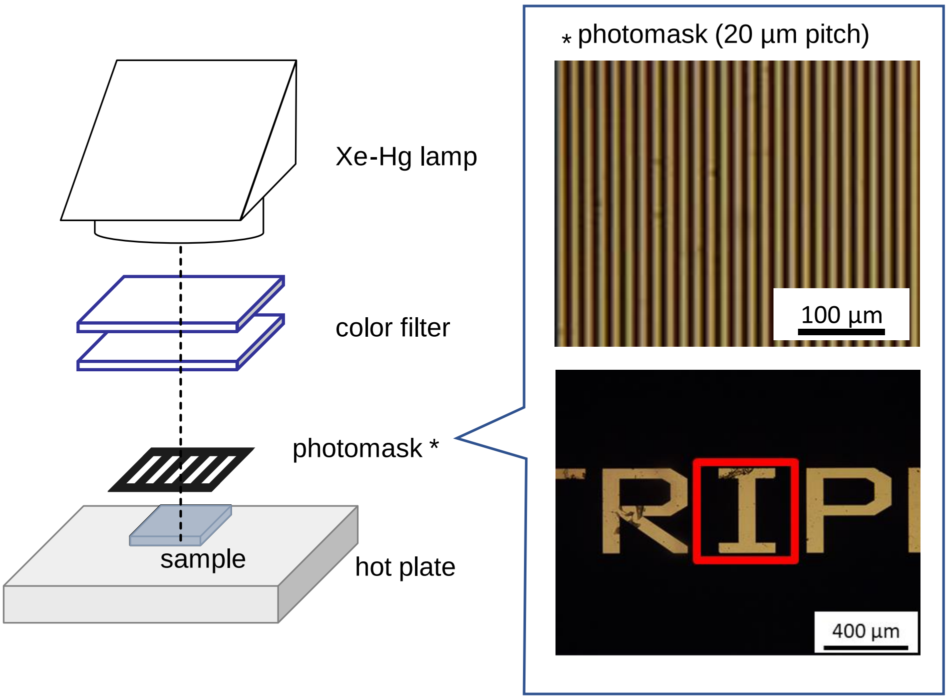

Figure S11. Experimental setup of UV light irradiation at 90 °C.
